# The Impact of Environmental Regulation on the Location of Pollution-Intensive Industries in China under Agglomeration Effect

**DOI:** 10.3390/ijerph18084045

**Published:** 2021-04-12

**Authors:** Yinhao Wu, Shumin Yu, Xiangdong Duan

**Affiliations:** 1 Institute of Economics and Management, Henan Agricultural University, Zhengzhou 450046, China; yhwu@henau.edu.cn; 2 Archival Research Centre, Henan Agricultural University, Zhengzhou 450001, China; dxd0105@henau.edu.cn

**Keywords:** environmental regulation (ER), agglomeration effect, economic development level, location of pollution-intensive industries (PIIs), influence mechanisms, moderated mediation model

## Abstract

Pollution-intensive industries (PIIs) have both scale effect and environmental sensitivity. Therefore, this paper studies how environmental regulation (ER) affects the location dynamics of PIIs under the agglomeration effect. Our results show that, ER can increase the production costs of pollution-intensive firms (PIFs) by internalizing the negative impact of pollutant discharge in a region, and thus, directly reduces the region’s attractiveness to PIFs. Meanwhile, ER can indirectly reduce the attractiveness of a region to PIFs by reducing the externality of the regional agglomeration effect. Moreover, these influences are regulated by the level of local economic development. Based on the moderated mediating effect model, we find evidence from the site selection activities of newly built chemical firms in cities across China. The empirical test shows that compared with 2014, the proportion of the direct effect of ER to the total effects significantly decreased in 2018, while the proportion of indirect effects under the agglomeration effect increased significantly. Our findings provide reference for the government to design effective environmental policies to guide the location choice of new PIFs.

## 1. Introduction

Sustainable development and increasing awareness of risks, resulting from environmental changes, have become the main incentives for governments to formulate regional and spatial development strategies [1]. The environmental dimension of spatial planning has been tested by a series of regional development theories and practices over the past decade [2,3,4]. The micro-manifestation of the regional development concept is the industrial space reconstruction, based on environmental constraints under the modern economic growth pattern, which is increasingly manifested as the spatial agglomeration of economic activities [5]. Therefore, it is of great significance to study the relationship between environmental regulation (ER) and industrial spatial dynamics under the agglomeration effect.

The spatiotemporal dynamics of pollution-intensive industries (PIIs) have attracted wide attention. On the one hand, local governments attach great importance to PIIs due to its huge economic development opportunities. On the other hand, PIIs are widely criticized by the public because of environmental and ecological problems caused by them [6,7]. It has become common practice to reduce the negative effects of pollution by resorting to environmental policies. The theoretical basis for this behavior is ‘pollution haven hypothesis’ (PHH). The PHH points out that strict ER will increase the compliance costs of polluting firms, thereby inhibiting the development of PIIs [8].

However, PIIs are also characterized by imperfect competition and increasing returns to scale [9]. Therefore, their location selection behavior is not only affected by ER, but also susceptible to the impact of the agglomeration economy. Therefore, the agglomeration economy should be taken into account when studying the location choice of PIIs with ER [9,10]. By realizing this, this paper aims to study the impact of ER on the location of PIIs from the perspective of the agglomeration economy. This would make up for the shortcomings of current research where the relationship between ER and the spatiotemporal dynamic of PIIs are studied without considering the agglomeration effect.

The chemical industry is a typical PIIs and one of the most important basic industries in the national economy. In 2018, the global chemical industry provided more than 120 million jobs and approximately US$5.7 trillion of output [11]. China is the largest chemical-manufacturing country in the world, and its chemical output value accounts for 40% of the global total [12]. However, the production and distribution of chemical products is dangerous and often poses a great threat to the surrounding ecological environment and safety, resulting in a general aversion to the chemical industry [6]. In recent years, the spatial layout of the chemical industry, especially the newly built chemical firms, has attracted great attention from the Chinese government. Therefore, this paper takes the spatiotemporal evolution of newly built chemical enterprises on the scale of Chinese cities as a study case to investigate the impact mechanism of ER on the site selection of new pollution-intensive firms (PIFs) under the agglomeration effect.

## 2. Literature Review

In response to the serious threats to public health posed by high pollution emissions, the Chinese government implemented strict environmental controls measures [13]. Therefore, designing of an effective ER has become one of the key challenges faced by PIIs [14,15,16,17]. To solve this problem, it is critical to clarify the influence mechanism and transmission path of ER on the location of PIFs. Scholars generally believe that ERs affect the location choice of PIFs by changing regional comparative advantages [18,19,20]. However, no consensus has been reached in both theoretical and empirical studies [7,17].

In theoretical studies, pollution haven hypothesis (PHH) argues that strict ER is detrimental to the creation of new PIFs because they increase the compliance costs of firms [21]. In contrast, the Porter hypothesis (PH) states that properly-designed ER can promote new polluting firms, as they can catalyze innovations that, to some extent, offset the compliance costs of firms, and even generates new regional competitive advantages [22].

In empirical studies, the existing research conclusions regarding the impact of ER on the location selection of polluting firms can be classified into three categories, being significant negative correlation [23,24,25], non-significant negative or even positive correlation [26,27,28], and nonlinear correlation [14,29,30]. For example, Li et al. (2017) [31] found that foreign chemical pharmaceutical plants in China tend to invest more in regions with loose ER. However, Ederington et al. (2005) [32] concluded that PIFs are relatively fixed, so they have a certain tolerance to an increase in environmental costs. Similarly, Kikpartick and Shimamoto (2008) [33] confirmed that foreign direct investment in Japan’s chemical industry and four other polluting industries is more likely to occur in regions with stricter ER. From a different perspective, Zhou et al. (2017) [17] argued that the influence of ER on the spatiotemporal dynamics of Chinese polluting firms is nonlinear, and that PH and PHH can coexist.

As a new economic geography emerges, the agglomeration economy has received some attention with respect to its impact on the location distribution of PIFs. PIFs are generally characterized by imperfect competition and increasing returns to scale [9], and their location selection behavior is, not only affected by ER, but also more susceptible to the agglomeration economy. Therefore, the agglomeration economy should be taken into account when studying polluting firms’ location choice with ER [9,10]. By realizing this, some research has studied the impact of ER from the perspective of agglomeration economy. Verbeke and Cleraq (2006) [10] confirmed that the spatial-temporal pattern of PIIs is affected by both ER and agglomeration economy, and that there may be an income effect. Zeng and Zhao (2009) [9] proved that the agglomeration economy plays a key role in the relationship between ER and the location distribution of PIFs through a theoretical analysis. Subsequently, Wagner and Timmins (2009) [34] provided further empirical support for the findings of Zeng and Zhao (2009) [9]. The most recent related research comes from Wu et al. (2020) [7] and Pang et al. (2019) [35]. The former discussed the threshold effect of agglomeration economy with respect to the relationship between ER and the location of new chemical firms. The latter explored the economic threshold of effective ER using China as the study context.

Based on the existing research, this paper tries to better understand how ER changes regional attraction by taking into account the effect of the agglomeration economy on the location choice of polluting firms with environmental restrictions. More specifically, this paper aims to decompose the impacts of ER on the location of polluting firms into different types, i.e., direct effects and indirect effects. In our setting, the agglomeration economy is regarded as an intermediary variable when we analyze the indirect effects. Meanwhile, the regional economic development level is included in our model as a moderating variable.

The rest of this article is organized as follows. Section 3 proposes a theoretical framework to analyze the impact mechanism of ER on the location choice behavior of new PIFs under the agglomeration effect and proposes several relevant research hypotheses. In Section 4, based on the polluting enterprise database established in this paper, we conduct a visual analysis of the spatiotemporal pattern of start-up pollution-intensive enterprises at the prefecture-level city scale in China. Section 5 introduces a moderated mediation model to empirically test the theoretical analysis and relevant hypotheses presented in Section 3. Finally, Section 6 concludes the paper and discusses possible future extensions.

## 3. Research Framework and Hypothesis Development

**Figure 1:**
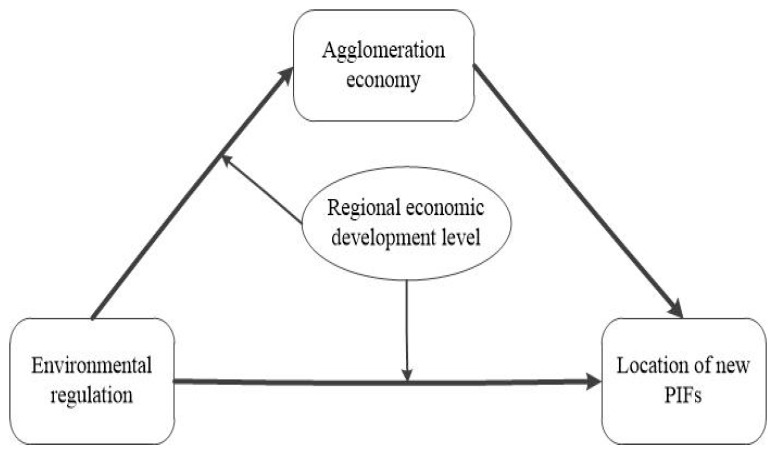
Influence mechanism of ER on the location of new PIFs under agglomeration effect.

We propose a moderated mediation effect analysis framework (Figure 1). By taking the agglomeration economy and the regional economic development level as as the intermediary variable, and the moderating variable, respectively, this paper explores the impact mechanism showing how ER affects the site selection of new PIFs under agglomeration effect.

### 3.1. ER and Site Selection of New PIFs

First, ER internalizes a firm’s environmental impact into its own costs [14,26]. As a consequence, the firm’s production cost increases and it becomes less competitive in the market. This decreases the willingness of new firms to enter. Secondly, from a regional perspective, strict ER distorts the spatial pattern of economic development. This distortion causes a disadvantage for enterprises with stricter ER in regional competition for attracting new enterprises and creating new jobs. Eventually, both the employment rate and the overall productivity in the region decline, and firms are forced to leave. Therefore, following first hypothesis is established:

**Hypothesis** **1.** *ER inhibits the establishment of new PIFs, and the working path includes direct and indirect effects.*

### 3.2. The Mediating Effects of Agglomeration Economy

In terms of ER, people’s perceptions are generally more closely associated with the entire industrial agglomeration than with the individual firm [36,37]. Therefore, the effect of ER on the agglomeration economy is also greater and more obvious. At the same time, the agglomeration economy, whether localized or urbanized, contributes to a stimulation of new industrial firm formation [38,39,40]. In other words, ER affects the regional economic agglomeration level, changes the regional attraction, and ultimately impacts the location selection strategy of new PIFs. Therefore, we formulated the following second hypothesis:

**Hypothesis** **2.** *The agglomeration economy plays a mediating role between ER and the location choice behavior of new PIFs.*

### 3.3. The Moderating Effects of Economic Development Level

The implementation of ER largely depends on the intentions of local governments, which are closely related to the level of local economic development [35]. In the early and middle stages of industrialization of developing countries [41], local offices are more inclined to prioritize economic development. However, both the environmental Kuznets curve and the underlying environmental demand theory confirmed that people’s demand for environmental public goods increases with the economic development level [42]. Then it can be summarized that the level of local economic development is a factor that can adjust the effect of ER on both newly built PIFs and the agglomeration economy. Therefore, we propose:

**Hypothesis** **3.** *The level of local economic development plays a moderating role in the relationship between ER and the location choice behavior of new PIFs.*

## 4. Data Source and New Chemical Firms’ Location Dynamics in China

### 4.1. Data Sources

Dependent Variable: As shown in Table 1, Y is the dependent variable. N represents the number of new chemical enterprises newly registered and established in each prefecture-level city in China each year. The original data were retrieved by the authors from official Chinese websites such as “National Enterprise Credit Information Publicity System” and “Credit China”. The data were supplemented by consulting the “Global Enterprise Database from Wind Information.” To the best of our knowledge, the database built in this paper, the “Database of Newly built Chemical Enterprises in Each Prefecture-level in China (2014–2018)” is the late stand most complete chemical enterprise database in China.

Independent Variables: First, researchers have conducted significant research on the measurement indicators and methods of ER. However, a unified measurement standard has not yet been formed [43,44]. Although there are more, or less, problems with different measurement indicators and methods, the multidimensionality and comparability of measurement indicators are the two key factors [44,45]. Based on the ideas of Keller [44] and Brunel [45], we construct the environmental regulation index measurement system and method. When measuring the intensity of local ER, it is necessary to simultaneously consider the pollution emission efficiency and the industrial structure characteristics of different regions. Therefore, this paper constructs a composite index that includes local pollution emission efficiency and local industrial structure characteristics. It should be noted that this article only uses this indicator to represent the intensity of regional ER, and does not pay attention to the deterministic impact on climate. The calculation formula is as follows:

(1) $$ER_{it}=\underset{j}{\sum }\frac{1}{PI_{itj}\times ST_{it}}=\frac{1}{PI_{it1}\times ST_{it}}+\frac{1}{PI_{it2}\times ST_{it}}+\frac{1}{PI_{it3}\times ST_{it}}$$

(2) $${\mathrm{PI}}_{itj}=\frac{EM_{itj}}{Y_{it}}$$

*EM_itj_* represents the total emission of pollutant *j* in region *i* in year *t*; *Y_it_* represents the total industrial output value of region *i* in year *t*. PI represents the intensity of pollution emissions, which is represented as the amount of pollutants emitted per unit of industrial output value. *PI*_1_, *PI*_2_, and *PI*_3_ represent the emission intensity of industrial wastewater, industrial sulfur dioxide, and industrial smoke (powder) dust, respectively. In the chemical enterprises industry, the main pollutant, include industrial wastewater, industrial sulfur dioxide and industrial smoke (powder) dust. In fact, there are various pollutant species contained in industrial wastewater or industrial smoke (powder) dust. Take industrial wastewater as an example, it contains pesticides, volatile organic compounds (VOCs) and heavy metals. Unfortunately, it is difficult to obtain data on the emissions of pollutants, such as pesticides, volatile organic compounds, and heavy metals. In particular, the emissions data on the city scale are seriously missing. Consequently, we use the industrial wastewater as the whole to represent the pollution emissions. Actually, pesticides, VOCs and other pollutants produced by the chemical industry, on the one hand, are mainly discharged in the form of wastewater. On the other hand, the statistics of wastewater discharge on the municipal scale in China is relatively transparent and detailed. *ST* stands for industrial structure, that is, the proportion of the output value of pollution-intensive industries. The letters *t* and *i* represent the year, and city, respectively.

Second, at present, there is still controversy about which forms of economic agglomeration, such as localization versus urbanization economies, are more attractive to enterprises [46,47,48]. However, there is no doubt that both forms can affect the spatiotemporal dynamics of firms through positive externalities [49]. Obviously, industrial agglomeration districts include both forms of agglomeration. In addition, the Chinese government has required new chemical firms to enter chemical industry zones or industrial zones since 2010. Therefore, in this paper, the location entropy of those provincial and national industrial parks with the chemical industry as the leading industry or one of the leading industries is used to capture the level of economic agglomeration in the region. The calculation formula is as follows,

(3) $$AE_{it}=\frac{S_{it}/A_{it}}{S_{t}/A_{t}}$$

where *S* represents the land area of the provincial and national industrial parks with the chemical industry as the leading industry or one of the leading industries. *A* represents the land area, and *i* and *t* represent prefecture-level city, and year, respectively.

Finally, considering factors such as local traffic conditions and labor costs will also influence the location decisions of enterprises, we included them in the model as control variables [38,50,51].

### 4.2. Dynamics of the Location of New Chemical Firms in China

**Figure 2:**
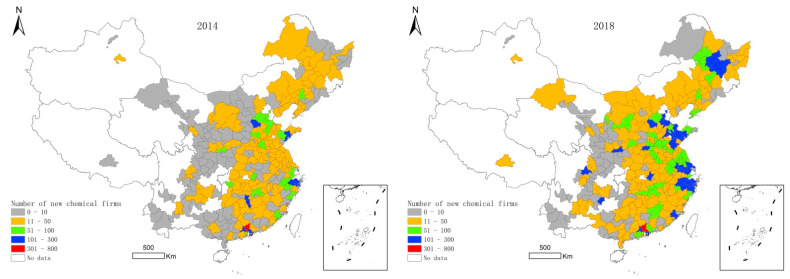
Location dynamics of new chemical firms in prefecture-level cities in China.

We use ArcGIS to visually express the spatiotemporal distribution of newly built chemical enterprises in various cities in the new era of China (2014–2018) and choose the two representative time points of 2014 and 2018 to perform a detailed analysis (Figure 2).

According to Figure 1, from 2014 to 2018, the total number of new chemical enterprises in China increased considerably, from 6574 to 11,659, representing an increase of over 77%. From the spatial perspective, new chemical enterprises were mainly concentrated in the eastern and central regions of China, with the fewest in the western region. Among them, the areas with the largest number of new chemical enterprises include the Bohai Rim Economic Belt, the Yangtze River Delta Economic Belt, the Pearl River Delta Economic Belt, and the “Changsha-Yichun-Jiujiang” critical band. However, in the past five years, the central region has seen the fastest increase in the number of new chemical firms, followed by the western and eastern noncoastal regions, with the eastern coastal region experiencing the lowest growth rate. These trends reflect a notable change in the spatial preferences of new Chinese chemical firms from 2014 to 2018. The central, western and eastern noncoastal regions are increasingly the first choice for newly built chemical enterprises. This may be closely related to a series of industrial transfer policies, based on environmental protection measures, implemented by the new Chinese government since 2014. In other words, ER has changed the spatial decision-making behavior of newly built enterprises in China.

## 5. Empirical Analysis

We performed a conditional process analysis, based on model 8, using the SPSS macro program developed by Hayes (2013) [52], and use bootstrapping to conduct a significance test on the regression coefficients of the variables [53]. The empirical test is divided into two parts. The first part is based on the simple mediating effect model to test the intermediary effect of agglomeration economy on the site selection of new chemical firms while controlling for regional traffic and labor costs. The second part is based on the moderated mediating effect model and tests the moderating mediating effect while controlling for regional traffic and labor costs. In addition, due to the time delay in the influence of the explanatory variables on the site selection of new chemical firms, endogeneity may exist between some explanatory variables and the explained variables, so we lag all explanatory variables by one stage.

### 5.1. Mediating Effect Test of Agglomeration Economy

From model 1A in Table 2, the regression coefficient of ER (*ER*) is −0.16 (β = −0.16), and the bootstrap test result is significant at the 5% level (95% *CI* ∈ [−0.14, −0,01]). Therefore, ER plays a significant role in predicting the agglomeration economy (*AE*); that is, the first half of the intermediary effect of the agglomeration economy on the distribution of newly built chemical enterprises is significant. At the same time, the prediction effect of the agglomeration economy on the location selection of new chemical firms (*lnY*) in model 1B is also highly significant (β = 0.32; 95% *CI* ∈ [0.18, 0.45]); that is, the influence of the agglomeration economy on the second half of the mediating effect on the new chemical firms’ distribution is significant. This finding proves that the agglomeration economy plays a significant mediating role in the relationship between ER and the location selection of new chemical firms and confirms hypotheses 1 and 2.

In addition, according to model 1B, it is easy to calculate the direct effect and total effect of ER on the location selection of new chemical firms as approximately −0.05, and −0.101, respectively. The direct effect of ER on the location of new chemical firms, namely, the mediating effect of the agglomeration economy, is approximately −0.051. The direct and indirect effects of ER on the location selection of new chemical firms account for 49.5%, and 50.5% of the total effects, respectively. That is, without considering the adjustment effect of economic development level (*lnAGDP*), the direct and indirect effects of ER on the spatial distribution of newly built chemical firms are basically equal.

### 5.2. Moderated Mediating Effect Test

We add a moderating variable, the regional economic development level, to model 1 to further test the moderated mediating effect. The results of model 2A show that the regression coefficient of the interaction term of ER and local economic development level (ER*lnAGDP) is significant at the 5% level (β = 0.11; 95% *CI* ∈ [0.11, 0.71]). This preliminarily confirms that the level of economic development plays an important mediating role in the relationship between ER and the agglomeration economy. Namely, the level of economic development effectively regulates the first half of the mediating effect of ER on the site selection of new chemical firms. Moreover, the regression coefficient of the interaction term is significantly positive, indicating that in developed regions, the negative impact of ER on the agglomeration economy is small. Second, the regression coefficient of the interaction term of ER and the local economic development level in model 2B is also significant (β = −0.26; 95% *CI* ∈ [−0.70, −0.19]). This result demonstrates that the economic development level also plays a significant negative mediating role in the direct effect of ER on the site selection of new chemical enterprises, confirming hypothesis 3. In short, at a higher level of economic development, the direct effect of ER on the site selection of new chemical firms is greater; that is, the exclusion effect is stronger.

**Figure 3:**
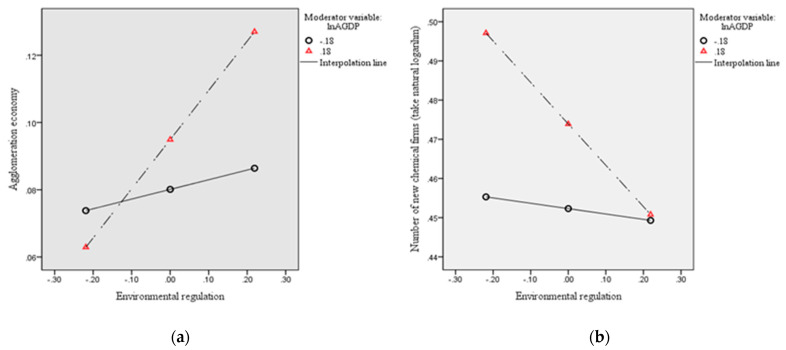
Moderating role of the economic development level (simple slope analysis). (**a**) Simple slope analysis of regions with high levels of economic development; (**b**) Simple slope analysis of regions with low levels of economic development.

To more intuitively describe the regulatory effect of economic development level, we draw a schematic diagram of the regulatory effect based on a simple slope analysis (Figure 3). According to Figure 3a, in regions with high economic development level (*lnAGDP* = M + 1SD), ER has a significant positive effect on the agglomeration economy; in regions with low economic development level (*lnAGDP* = M − 1SD), the positive effect is not significant. This finding shows that with the improvement in the local economic development level, ER plays a more important role in predicting the agglomeration economy. Similarly, according to Figure 3b, in regions with a high level of economic development (*lnAGDP* = M + 1SD), ER has a significant negative prediction effect on the site selection of new chemical firms. While in regions with a low level of economic development (*lnAGDP* = M − 1SD), the prediction effect is not significant. These results show that with the improvement in the local economic development level, the negative prediction effect of ER on the site selection of new chemical enterprises is gradually strengthened.

Finally, we also measure the mediation effect of the agglomeration economy under different economic development levels (Table 3). With the improvement in economic development levels (regulatory variable), the mediating effect of the agglomeration economy on the relationship between ER and site selection of newly built chemical enterprises is also significantly enhanced. As the local economic development level increases, the effect of ER on the agglomeration economy has a downward trend.

### 5.3. The Influence of ER on Site Selection of Newly Built Chemical Enterprises in Different Years

We use the same method to examine the mediating effect of the agglomeration economy and the moderating effect of the local economic development level in 2018. We further confirm the influence mechanism and transmission path of ER on the location of newly built chemical enterprises (Table 4). At the same time, the direct and indirect effect of ER on the site selection of new chemical firms (i.e., the mediating effect of the agglomeration economy) is also quantitatively measured.

The comparison between Table 2 and Table 4 reveals that the influence mechanism and transmission path of ER on the site selection of newly built chemical enterprises are consistent. First, ER can directly reduce the number of new chemical firms and reduce the region’s attractiveness to new chemical firms by inhibiting regional economic agglomeration. Second, the level of local economic development plays a significant regulatory role. For example, a higher level of economic development reduces the negative effect of ER on the agglomeration economy, but it significantly improves the exclusion effect of ER on new chemical enterprises. Finally, the direct effect of ER accounted for 49.5% of the total effect in 2014, but this proportion dropped to approximately only 10% in 2018. In other words, compared with 2014, the ER in 2018 focused more on indirectly changing the location decision of new chemical enterprises by adjusting the level of regional economic agglomeration.

It should be noted that the focus of this empirical test is on the impact of ER on the location of new chemical firms. The final location of new chemical firms depends on comprehensive trade-offs among various influencing factors.

## 6. Conclusions and Discussion

### 6.1. Conclusions

Incorporating environmental and agglomeration factors into the firms’ location decision is a complex process. This paper discusses the mechanisms and effects of ER on the site selection of new PIFs under the agglomeration effect. Through the proposed transmission mechanism analysis framework of “environment regulation→regional competitive comparative advantage→location of new PIFs,” we tried to open the “black box” of how ER affects the site selection of new PIFs under the agglomeration effect. We found that ER can internalize pollutant discharge and increase the production costs of polluting firms in the region, and thus, directly reduce the attractiveness of the region to polluting firms. At the same time, the ER can also indirectly reduce the attractiveness of the region to polluting firms by reducing the degree of regional economic agglomeration (reducing the externality of regional agglomeration). Moreover, these influences are regulated by the level of local economic development in the transmission process. Based on the moderated mediating effect model, we also found evidence from the site selection activities of newly built chemical enterprises in cities across China.

### 6.2. Discussion

In dialogue with the existing literature, we respond to, and confirm, the scholarly view that the role of the agglomeration economy should be emphasized when studying how ER affects the spatial distribution of PIFs [9,34,54], and we analyze the specific path through which these effects are transmitted. The present study clearly proposes and confirms that the effect of ER, includes not only direct effects [18], but also indirect effects. The regulation effect of the economic development level or the agglomeration economy on ER, such as the threshold effect [34], is verified in this paper as well. In addition, this paper constructs a database of Chinese new chemical firms. The database includes information on newly established chemical firms in all Chinese cities since 2013. To some extent, this completes the existing empirical research data, for example, most of the data used in study were before 2013. In fact, since 2013, China has implemented the most stringent environmental protection policy in history, which has caused significant changes in the spatial preferences of PIFs represented by chemical firms.

Although our research provides new ideas for discussing how ER affects the location of newly built PIFs under the agglomeration effect, there is still room for improvement. For example, industry and spatial heterogeneity may also change the effect of ER [55,56,57]. Unfortunately, due to the limitations of subdivided industry data, we have to make a trade-off between the level of geographic and industry detail. Therefore, it could be interesting to study the relationship between ER and the location of newly built PIFs, based on subdivided industries (e.g., three-digit industry defined by SIC code) and enterprise characteristics (e.g., enterprise size and ownership). In addition, using computer simulation technology to simulate the spatial evolution of new PIFs under different environmental regulatory intensities, and to evaluate the impact of ERs on the spatial distribution of new PIFs will be another important direction for future research.

## Figures and Tables

**Table 1:** Calculation methods, sources of variables and their basic statistical characteristics.

Variable Set	Variable Definition	Calculation Methods (Dimension)	Data Sources	Basic Statistics				
				Mean	Std.	Min.	Max.	Obs.
Dependent variable	The number of new chemical firms in each city, *lnY*	Y = N + 1, N is the number of new chemical firms with registered capital of more than 1 RMB	★☆＊	3.069	1.158	0.000	6.571	562
Independent variables	Environmental regulation, *lnER*	$ER_{it}\;=\;\frac{1}{PI_{it1}\times ST_{it}}+\frac{1}{PI_{it2}\times ST_{it}}+\frac{1}{PI_{it3}\times ST_{it}}$ (dimensionless)	●@○	0.712	0.116	0.426	0.996	562
	Agglomeration economy, *AE*	$AE_{it}\;=\;\frac{S_{it}/A_{it}}{S_{t}/A_{t}}$ (dimensionless)	∆	0.719	1.568	0.000	14.776	562
Control variables	Economic development level, *lnAGDP*	Percapita gross national product, taking the natural logarithm (constant price, 100 M yuan)	● ※	10.624	0.605	9.037	12.050	562
	Traffic condition, *lnTC*	Highway freight volume, taking the natural logarithm (10 K tons)	●	9.048	0.874	6.324	17.013	562
	Labor wages, *lnLW*	Wages of on-the-job employees, taking the natural logarithm) (constant price, yuan/year)	● ※	10.550	0.212	9.876	11.233	562

★ National Enterprise Credit Information Public System. http://www.gsxt.gov.cn/ (accessed on 7 February 2021); ☆ Credit China. https://www.creditchina.gov.cn/ (accessed on 7 February 2021); ＊ Global Enterprise Database from Wind Information; ● National Bureau of Statistics of China. China City Statistical Yearbook (2013 and 2017); @ National Bureau of Statistics of China. China Environmental Statistical Yearbook (2013 and 2017); ο National Bureau of Statistics of China. China Industrial Statistical Yearbook (2013 and 2017); Δ China Development Zone Audit Catalog (2018 Edition); ※ Statistical Yearbook of each prefecture-level city of China (2013 and 2017).

**Table 2:** Regression results of the moderated mediation model (Bootstrap = 5000) (2014).

	Mediation Effect Test						Moderated Mediation Effect Test					
	*AE* Model 1A			*lnY* Model 1B			*AE* Model 2A			*lnY* Model 2B		
	β	*t*	95% *CI*	β	*t*	95% *CI*	β	*t*	95% *CI*	β	*t*	95% *CI*
*ER_it−_*_1_	−0.16 **	−2.15	[−0.14, −0.01]	−0.05 **	−2.29	[−0.13, −0.04]	−0.08 ***	−2.65	[−0.16, −0.02]	−0.06 **	−2.04	[−0.07, −0.02]
*AE_it−_*_1_				0.32 ***	4.57	[0.18, 0.45]				0.30 ***	4.61	[0.18, 0.46]
*ER_it−_*_1_ × *lnAGDP_it−_*_1_							0.11 ***	2.72	[0.11, 0.71]	−0.26 ***	−2.35	[−0.70, −0.19]
*lnAGDP_it−_*_1_							0.32 ***	4.61	[0.18, 0.46]	0.04	0.91	[−0.05, 0.13]
*lnTC_it−_*_1_	0.52 ***	3.78	[0.09, 0.32]	0.21 ***	9.59	[0.42, 0.63]	0.14 ***	3.03	[0.05, 0.23]	0.11 ***	9.43	[0.41, 0.63]
*lnLW_it−_*_1_	−0.03	1.08	[−0.04, 0.10]	−0.05	−0.62	[−0.07, 0.16]	−0.07	−0.92	[−0.28, 0.04]	−0.03	−0.65	[−0.10, 0.16]
*cons*	0.04 **	2.06	[0.00, 0.07]	0.17 ***	4.26	[0.09, 0.25]	−0.07 **	−2.20	[−0.13, −0.01]	0.15 ***	4.11	[0.07, 0.22]
*R* ^2^	0.14			0.38			0.15			0.44		
*F*	15.26 ***			41.46 ***			9.88 ***			28.26 ***		

Note: The bootstrap test shows that under the 95% confidence interval, if the interval formed by *CI* does not contain the value 0, the test result is significant at the 5% level (the same below). ** *p* < 5%, *** *p* < 1%.

**Table 3:** Mediating effects at different economic development levels.

	Economic Development Level	Effect Value	Boot SE	Boot 95% *CI*
The mediating effect of AE (with moderating variable)	Effect1 (*lnAGDP* = M − 1SD)	0.009	0.012	[−0.02, 0.03]
	Effect2 (*lnAGDP* = M)	0.028	0.011	[0.01, 0.05]
	Effect3 (*lnAGDP* = M + 1SD)	0.047	0.020	[0.01, 0.09]

**Table 4:** Regression results of the moderated mediation model (Bootstrap = 5000) (2018).

	Mediation Effect Test						Moderated Mediation Effect Test					
	*AE*			*lnY*			*AE*			*lnY*		
	β	*t*	95% *CI*	β	*t*	95% *CI*	β	*t*	95% *CI*	β	*t*	95% *CI*
*ER*_*it*−1_	−0.23 ***	−2.63	[−0.12, −0.01]	−0.01 **	−2.17	[−0.08, −0.01]	−0.14 **	−2.44	[−0.21,−0.02]	−0.03 **	−1.99	[−0.06, −0.01]
*AE*_*it*−1_				0.39 ***	3.94	[0.26,0.77]				0.32 ***	4.51	[0.23, 0.56]
*ER*_*it*−1_×*lnAGDP*_*it*−1_							0.15 **	2.10	[0.17, 0.33]	−0.28 ***	−2.47	[−0.78, −0.13]
*lnAGDP*_*it*−1_							0.36 ***	6.09	[0.24, 0.48]	0.01	0.34	[−0.06, 0.04]
*lnTC*_*it*−1_	0.61 ***	8.03	[0.01, 0.12]	0.27 ***	5.16	[0.54, 0.82]	0.41 ***	5.77	[0.10, 0.43]	0.06 **	2.01	[0.05, 0.23]
*lnLW*_*it*−1_	−0.01	−0.40	[−0.07, 0.04]	−0.05	−1.25	[−0.04, 0.12]	−0.02	−0.71	[−0.03, 0.29]	−0.03	−0.34	[−0.13, 0.09]
*cons*	0.04 **	2.06	[0.00, 0.07]	0.04 **	2.06	[0.04, 0.83]	−0.05	0.13	[−0.13, −0.01]	0.14 ***	6.17	[0.08, 0.62]
*R* ^2^	0.03			0.35			0.07			0.43		
*F*	6.71 ***			37.20 ***			4.32 ***			34.70 ***		

** *p* < 5%, *** *p* < 1%.

## Data Availability

The data presented in this study are available on request from the corresponding author. The data are not publicly available due to the author and the co-owner of the data have signed a license agreement.
